# Arabinogalactan-Functionalized Gold Nanoparticles Demonstrated Remarkable Anticancer Therapeutic Effect against Hepatocellular Carcinoma

**DOI:** 10.1021/acs.molpharmaceut.5c00598

**Published:** 2025-10-13

**Authors:** Vasumathi R, Maya P Shetty, Suvalakshmi S, Revathi P Shenoy, Srinivas Mutalik, Sanjay Bharati

**Affiliations:** † Department of Nuclear Medicine, Manipal College of Health Professions, 76799 Manipal Academy of Higher Education (MAHE), Manipal 576104, Karnataka, India; ‡ Department of Biochemistry, Kasturba Medical College, Manipal Academy of higher education, Manipal Academy of Higher Education (MAHE), Manipal 576104, Karnataka, India; § Department of Pharmaceutics, Manipal College of Pharmaceutical Sciences, Manipal Academy of Higher Education (MAHE), Manipal 576104, Karnataka, India

**Keywords:** Hepatocellular carcinoma, active targeting, passive targeting, gold nanoparticles, asialoglycoprotein receptors

## Abstract

The treatment and prognosis of cancer remain highly challenging due to its complex and multifaceted nature. In the paradigm of nanomedicine, nanotechnology has gained huge attention in cancer therapeutics. The unique physicochemical properties offered by gold nanoparticles (AuNPs) in targeted drug delivery and anticancer therapy favor their implications in cancer therapeutics. However, surface modification, particularly with polysaccharides, can further optimize the function of gold nanoparticles. Among others, the advantages offered by arabinogalactan (AG) make it a suitable candidate for coating gold nanoparticles. The present study reported the anticancer therapeutic potential of AG-AuNPs against liver cancer. The synthesized AG-AuNPs were characterized for UV–visible spectrum, hydrodynamic size, zeta potential, core size, bonding and functional groups, crystalline nature, and elemental composition. The compound was then evaluated for its stability in biological fluids followed by *in vivo* biodistribution and anticancer therapeutic potential in rodent model of hepatocellular carcinoma (HCC). The chemical characterization of AG-AuNPs revealed a successful coating of AG onto AuNPs. The nanoparticles were stable in serum for up to 24 h with no appreciable change in the size of the UV–visible spectrum. The *in vivo* biodistribution study demonstrated excellent selective accumulation of AG-AuNPs in liver tumors with 1.6 times higher uptake as compared to nontumor liver tissue. AG-AuNPs showed a significantly decreased tumor load and restoration of tissue histoarchitecture in the treatment group as compared to the untreated tumor group. Overall, this study suggested that AG-AuNPs demonstrated a good anticancer therapeutic effect for the targeted treatment of HCC.

## Graphical abstract

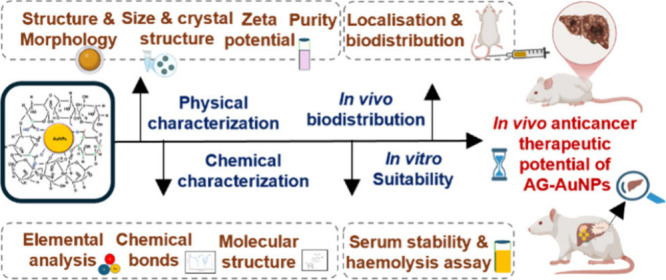

## 1 Introduction

Cancer is a major global health concern driven by highly heterogeneous processes making its treatment and prognosis highly challenging.^1^ Despite tremendous advancements in anticancer research, a safe and effective treatment strategy remains limited.^2^ Especially, the rising incidence, late diagnosis, and poor clinical outcomes of hepatocellular carcinoma (HCC) highlight its significance as a major public health concern accounting for approximately 758,725 deaths each year. Surgical resection, liver transplantation, transarterial chemoembolization (TACE), and systemic therapies are some of treatments of choice for HCC. However, certain limitations such as drug resistance, systemic toxicity, and high recurrence rate underscore the urgent need for development of innovative approaches to enhance drug delivery to tumors.^3^

Nanotechnology has emerged as a transformative paradigm in cancer therapeutics enabling precise delivery of therapeutic drugs to hepatoma cells while lowering the off-target accumulation and thus, systemic toxicity.^4^ Several classes of nanoparticles like carbon nanotubes, iron oxide nanoparticles, liposomes, dendrimers, etc. have been widely employed to overcome the limitations of current liver cancer therapies. Among others, gold nanoparticles are an attractive choice for cancer therapy due to their unique physicochemical properties.^5^ Their high surface area-to-volume ratio and ease of functionalization facilitate the conjugation of therapeutic agents and targeting ligands, enabling both active and passive targeting of tumor cells while sparing healthy tissues.^6^ Furthermore, the ability of AuNPs to induce cancer cell death through mechanisms such as reactive oxygen species (ROS) generation, disruption of cellular homeostasis, and activation of apoptosis makes them a versatile and effective anticancer agent.^7,8^ However, the therapeutic potential of AuNPs is largely influenced by their surface properties which govern their stability, bioavailability, and interaction in a biological system. Therefore, to further enhance their efficiency and safety, surface functionalization has emerged as a promising strategy.^9^

Several reports suggested that surface modifications using polysaccharides can be a suitable alternative strategy to harness the functional advantages offered by AuNPs.^10,11^ This is mainly due to the presence of functional groups in polysaccharides, such as hydroxyl groups that facilitate simpler chemical modifications thereby enhancing solubility, stability, and biological activity of AuNPs.^12^ In addition, its remarkable anti-inflammatory, antioxidative, and antitumor properties reported in literature underscore the importance of coating AuNPs with natural polysaccharides.^13,14^ Among different polysaccharides, arabinogalactan is a natural agent that has gained a great deal of attention for its chemical and biological properties. Its unique structural property enables it to efficiently interact with metal nanoparticles thereby ensuring uniform dispersion and enhanced *in vivo* stability.^15^ Several reports have suggested that polysaccharide-based AuNPs exhibit a significant effect on the inhibition of tumor metastasis.^16,17^ This can be primarily attributed to the ‘enhanced penetration and retention effect’, which facilitates tumor-targeted drug delivery.^18,19^ In addition, inherent anticancer properties of arabinogalactan including antiangiogenic and pro-apoptotic properties make it a suitable polysaccharide of choice for cancer treatment.^20^ Apart from this, arabinogalactan has also been widely employed for receptor-mediated targeting of asialoglycoprotein receptors (ASGPRs) for treatment of hepatocellular carcinoma (HCC).^21^ This targeted delivery is attributed to the higher binding affinity of arabinogalactan to ASGPRs, which are overexpressed in HCC.^22^ Thus, coating AuNPs with arabinogalactan can be a potential strategy for achieving effective anticancer therapy against HCC.

Therefore, in the present study, we report the development and assessment of AG-AuNPs for the treatment of HCC.

## 2 Materials and Methods

### 2.1 Chemicals and Kits

Arabinogalactan larch powder, gold­(III) chloride trihydrate, *N-nitrosodiethylamine (NDEA)*, and nitric acid were procured from Sigma-Aldrich Co. (St. Louis, USA). Liver function test kits were procured from Tulip Diagnostics (Santacruz, India). The remaining chemicals used in the study were sourced from local Indian firms and were of analytical grade.

### 2.2 Synthesis of Arabinogalactan-Coated Gold Nanoparticles

Arabinogalactan-coated gold nanoparticles were synthesized using the Turkevich method with some modifications.^23^ Briefly, 3% (w/v) arabinogalactan (AG) aqueous solution (10 mL) was prepared and heated for 5–10 min until it reached a temperature of 75–80 °C. 700 μL of 10 mM of hydrogen tetrachloroaurate (HAuCl_4_) was added dropwise to AG solution under continuous stirring at 1500–1800 rpm. The change in color from colorless to ruby red confirmed the formation of arabinogalactan-coated AG-AuNPs. The obtained AG-AuNPs were precipitated by using 4-fold ethanol and lyophilized to complete dryness.

### 2.3 Chemical Characterization of AG-AuNPs

#### 2.3.1 Ultraviolet–Visible Spectroscopy

UV–vis spectra of AG-AuNPs were obtained by using a UV–vis spectrophotometer (UV-2600 Shimadzu, Kyoto, Japan). The AG-AuNPs were scanned in the range of 200–1000 nm at a scan speed of 480 mm/min in a 1 mL quartz cuvette. Baseline-adjusted spectra were obtained and compared to a reference blank. The obtained spectra were assessed for changes in the intensity and wavelength in comparison to standards.

#### 2.3.2 Hydrodynamic Size and Zeta Potential

The mean hydrodynamic size and zeta potential of AG-AuNPs were analyzed by using dynamic light scattering (Nano series, Malvern Instruments Ltd., Worcestershire, UK). At backscatter angle of 90° and temperature of 25 °C, 0.25 mg/mL AG-AuNPs were analyzed with a fixed run time of 15 s per sample (n = 3). The size obtained was represented as an intensity average and expressed as the mean ± SD.

#### 2.3.3 Fourier Transform Infrared (FTIR) Spectroscopy

An FTIR spectrum of AG-AuNPs was obtained on an Attenuated Total Reflectance-Fourier Transform-Infrared (ATR-FTIR) spectrophotometer (IRSpirit, Shimadzu, Kyoto, Japan). The lyophilized samples were placed on sample holders and scanned in the frequency range (4000–400 cm^–1^). Each spectrum was baselined and normalized to adjust the optical characteristics. The obtained data were analyzed for the chemical bonds.

#### 2.3.4 Nuclear Magnetic Resonance (NMR) Spectroscopy

^1^H and ^13^C NMR spectra were performed to determine the molecular structure of AG-AuNPs. AG-AuNPs were dissolved in D_2_O and read on a 400 MHz high-performance NMR spectrometer (AV400 Bruker, US). ^1^H NMR and ^13^C NMR spectra were acquired for 16 and 4096 ns, respectively. The obtained NMR spectra were examined for variations in the functional groups at different carbon and proton atoms.

#### 2.3.5 X-ray Diffraction (XRD)

XRD measurement of AG-AuNPs was performed on an X-ray diffractometer (Rigaku, Japan) at 40 kV and 30 mA with Cu Kα radiation at a scan rate of 2.0°/min. The data obtained was normalized, baselined, and compared with standard Au (JCPDS card no. 4-0784). The obtained spectrum was analyzed for crystal type, size, lattice, composition, and phases.

#### 2.3.6 Energy-Dispersive X-ray (EDX) Spectroscopy

EDX was performed to determine the elemental composition of AG-AuNPs (percentage of Carbon, Oxygen, and Gold), using a SEM-EDX analyzer (Oxford Instruments X-MAX, Abingdon, UK). Lyophilized sample was placed in a sample suspension disc and sputtered with silver–palladium. Data acquisition was carried out at a resolution of 126.1 eV to ensure precise elemental detection and quantification. The resulting spectra were processed to calculate the percentage composition of each element present in the AG-AuNPs.

#### 2.3.7 Transmission Electron Microscopy (TEM)

TEM analysis was performed to determine the shape, core size, and agglomeration in AG-AuNPs using a transmission electron microscope (JEM-2100, JEOL Ltd., Tokyo). Briefly, the sample was placed on a microscope grid (copper support covered with carbon), and micrographs were obtained using a field emission gun operated at an accelerating voltage of 120 kV. The obtained data was analyzed for morphology and average core size using ImageJ software.

### 2.4 *In Vitro* Stability and Hemolytic Effect of AG-AuNPs

#### 2.4.1 Serum Stability Test of AG-AuNPs

The stability of AG-AuNPs was evaluated in rat blood serum as described by Foo et al., 2017.^24^ Briefly, AG-AuNPs dispersed in phosphate-buffered saline (PBS) were mixed with serum in a 1:1 volume ratio. Serum blanks served as a negative control. The change in the absorbance spectra of AG-AuNPs incubated for 3, 6, 12, and 24 h at 37 °C was monitored using UV–vis spectroscopy. In addition, the hydrodynamic size of AG-AuNPs (n = 3) after 24 h of incubation was also measured using a Zetasizer Nano ZS (Malvern Instruments, Malvern, UK).

#### 2.4.2 Hemolysis Assay

The hemolytic effect of the compound AG-AuNPs was evaluated prior to *in vivo* administration as per the method of Neun et al., 2011 with minor modifications.^25^ Briefly, freshly collected rat blood was subjected to centrifugation at 100 × g for 5 min. Further, the leukocyte-rich buffy coat was carefully removed by aspiration, and the erythrocyte fraction in the pellet was washed with 0.9% sodium chloride. The hemolytic potential of AG-AuNPs was assessed at concentrations of 0.5, 1, and 1.5 mg/mL, respectively. The percentage hemolysis obtained was compared with the negative control (Ca^2+^/Mg^2+^ phosphate buffer saline, pH 7.4) and the positive controls (1% triton X-100). The test was performed in triplicate, and data were represented as mean ± SD (n = 3).

### 2.5 *In Vivo* Biodistribution and Anticancer Therapeutic Potential of AG-AuNPs

#### 2.5.1 Animal Grouping and HCC Model Development

All animal experiments were performed after obtaining clearance from the institutional animal ethics committee (*IAEC/KMC/69/2022*) in accordance with guidelines of the committee for the purpose of control and supervision of experiments on animals (CPCSEA), India. Male Wistar rats of 250–300 g were housed in a central animal facility under controlled temperature (25 ± 2 °C), humidity (65–80%), and dark-light cycle (12 h). All animals were fed a standard pellet diet and clean drinking water *ad libitium*. Before initiating the experiment, all animals were acclimatized for a period of 1 week. A hepatocellular carcinoma (HCC) model was developed by administering *N-nitrosodiethylamine* (50 mg/kg b.w) intraperitoneally once a week for 14 weeks.^26^ After 14 weeks, the presence of HCC was confirmed by measuring the glypican-3 (GPC-3) levels in serum. Animals with 2-fold or more increase in GPC-3 levels as compared to Control group animals were considered positive for HCC and randomly segregated into Tumor group and Tumor + AG-AuNPs groups.

#### 2.5.2 *In Vivo* Biodistribution of AG-AuNPs

The *in vivo* biodistribution was performed to evaluate the localization of AG-AuNPs in various organs at 48 h postinjection as described by Bailly et al., 2019.^27^ Briefly, animals in Tumor + AG-AuNPs group (n = 3) were administered with AG-AuNPs (IV). After 48 h, animals were sacrificed and tissue samples like blood, heart, liver (tissue, tumor), spleen, kidney, lungs, and testis were collected. The biodistribution of AG-AuNPs (Au^3+^ ion) in all samples was analyzed using inductively coupled plasma mass spectrometry (ICP-MS).^28^ The sample preparation for ICP-MS analysis was performed as described previously by Sonavane et al., 2008.^29^ The total amount of AG-AuNPs in each tissue was expressed as μg/mg tissue and percent injected dose per gram of tissue (% ID/g).

#### 2.5.3 *In Vivo* Anticancer Therapeutic Potential of AG-AuNPs

After the development of the HCC model, Tumor group animals were randomly segregated as Tumor groups (n = 6) and Tumor + AG-AuNPs group (n = 6). Control group (n = 6) and AG-AuNPs group (n = 6) served as standard control and drug control groups. AG-AuNPs and Tumor + AG-AuNPs group animals received 1 mg/kg (0.1 mL) b. w. of AG-AuNPs intravenously, once every seventh day, for a period of 30 days. Control and Tumor groups were provided with no special treatment. After completion of the treatment period, all animals were sacrificed and the *in vivo* anticancer therapeutic effect of AG-AuNPs was assessed in terms of tumor statistics, dielectric properties, and histopathological changes.

#### 2.5.4 Tumor Statistics

The liver tissues/tumors were examined for gross morphological changes and total number of tumors. Based on size, the tumors were further classified as small (<3 mm) and big (≥3 mm) sized tumors. Tumor multiplicity was calculated by dividing the total number of tumors in each animal to the total number of animals having tumors.

#### 2.5.5 Dielectric Properties of Liver Tumors

The electrical conductivity of liver tissues/tumors was assessed using a two-pin silver electrode as described earlier.^30^ Briefly, the excised liver tissue/tumors were noted for their conductivity in the frequency range 4 Hz- 5 MHz using an impedance analyzer (Hioki-IM3570, Japan). The tumors were then classified as moderate and high degree of conductivity tumors.

#### 2.5.6 Histopathological Assessment of Liver Tumors

The histopathological changes in liver tissue/tumors were assessed using H & E staining according to standard laboratory procedures.^31^ Briefly, tissues were formalin-fixed (10% w/v) and embedded in paraffin wax. Five μM paraffin-embedded sections were mounted on a glass slide and stained with hematoxylin and eosin stain. The stained sections were then examined under a light microscope (Labomed, Lx-300, USA).

### 2.6 Statistical Analysis

Data were presented as mean ± SD. The Shapiro-Wilk test was applied to assess the normality, and Levene’s test was used to evaluate the homogeneity of variances. For intergroup comparisons one-way ANOVA followed by Tukey’s posthoc test was performed. Tumor multiplicity was analyzed using Student’s *t* test. A *p* ≤ 0.05 was considered statistically significant.

## 3 Results

### 3.1 AG-AuNPs Were Successfully Synthesized and Characterized Using a Modified Turkevich Method

AG-AuNPs were successfully synthesized using the Turkevich method. AG-AuNPs appeared ruby red in color indicating reduction of gold ions to gold nanoparticles. These results were further supported by the UV–Vis spectrum of AG-AuNPs, demonstrating a typical characteristic absorption peak at 542 nm indicating the formation of uniformly dispersed nanoparticles (Figure 1a).

**Figure 1:**
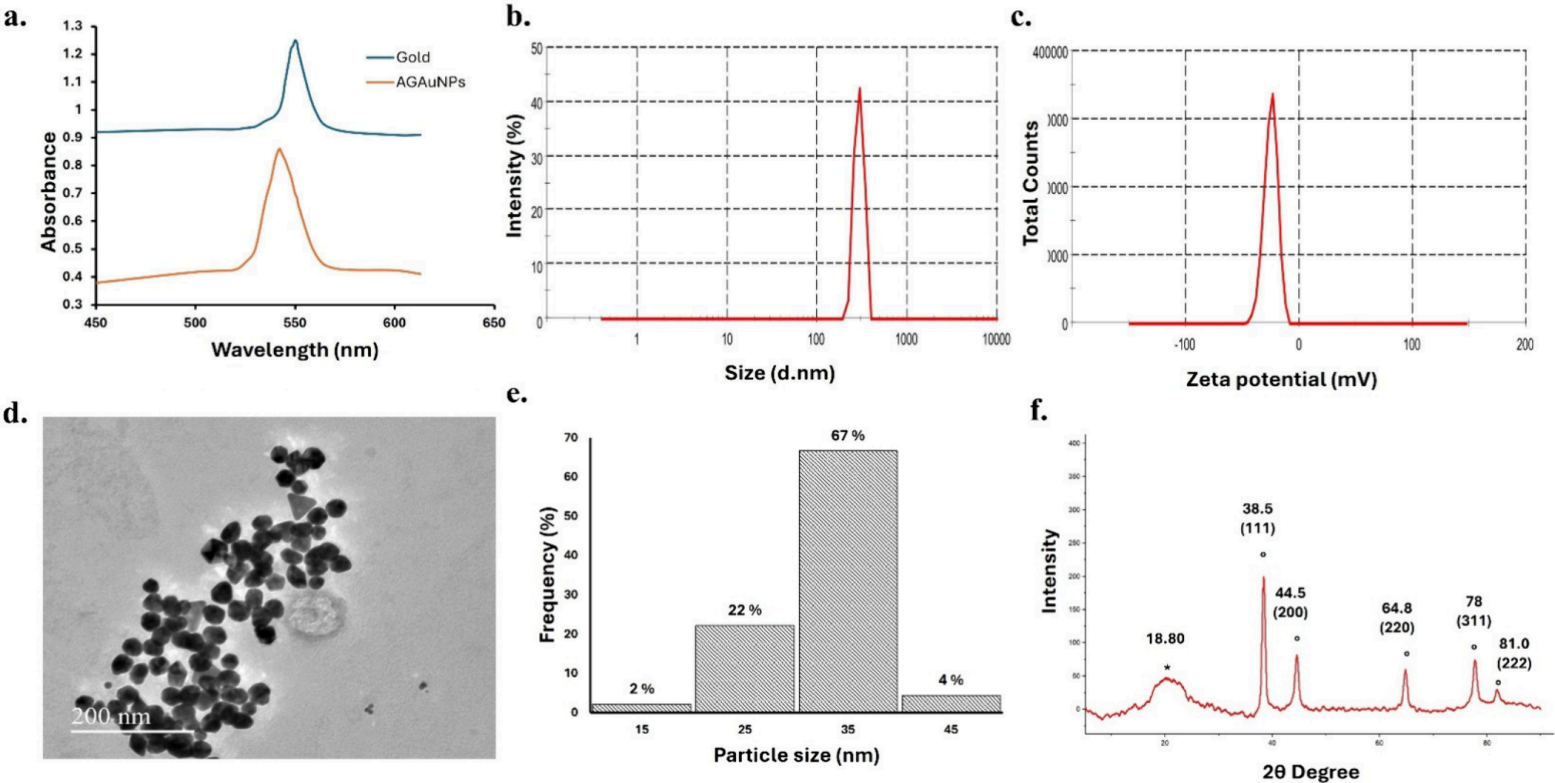
**Characterization of AG-AuNPs**. (a) UV-Spectrum of AG-AuNPs indicating shift in maximal absorption peak to 542 nm after AG coating. (b) Hydrodynamic size distribution of AG-AuNPs showing average diameter of 326.1 ± 2.7 nm (Scale: 0 to 1000 nm). (c) Zeta potential of AG-AuNPs showing negative surface charge (Scale: −200 to 200 mV). (d) High-resolution TEM images depicting showing AG-AuNPs were spherical in shape (Scale: 200 nm). (e) Particle size distribution histogram representing the frequency (%) of AG-AuNPs across different size ranges. (f) X-ray diffraction pattern of AG-AuNPs. The peaks of AG-AuNPs corresponding to the crystal planes of gold nanoparticles are marked as (°). The peak corresponding to arabinogalactan is marked as (*).

The average hydrodynamic size of AG-AuNPs was 326.1 ± 2.7 nm, with a PDI value of 0.25. These results indicated that AG-AuNPs were monodispersive and can be suitable for *in vivo* applications (Figure 1b).^32,33^ The zeta potential measurements revealed that AG-AuNPs had a negative charge of −22 mV which can be attributed to the presence of ionized functional groups from arabinogalactan, such as carboxylate (−COO^–^) and hydroxyl-derived species, on the nanoparticle surface (Figure 1c). The core size and shape of AG-AuNPs were further determined using TEM. TEM micrographs showed that AG-AuNPs were spherical in shape with relatively uniform size distribution (Figure 1d). The size distribution histogram indicated that 70% of the particles were in 30–40 nm range (n = 30) with mean size of 35.7 ± 2.5 nm (Figure 1e).

The crystalline properties and phases of AG-AuNPs were analyzed using XRD. The diffraction pattern of AG-AuNPs exhibited four distinct characteristic peaks at 2θ of 38.5°, 44.5°, 64.8°, and 78° that corresponded to the crystal planes (111), (200), (220), and (78), respectively. The typical XRD diffraction pattern of gold nanoparticles added further evidence on the formation of AG-AuNPs (Figure 1f) (JCPDS data number 04-0783 card and 4-0784 card). The crystalline size of AG-AuNPs determined using the Scherrer equation was 16.89 nm. These results indicated that AG-AuNPs had a face centered cubic (fcc) lattice structure and were crystalline in nature.

The FTIR spectrum of AG-AuNPs (Figure 2a), with the introduction of several new peaks to the distinctive peaks of AG, added further evidence of chemical bonding between AG and AuNPs. The AG FTIR spectrum showed absorption peaks at 3303 cm^–1^ and 1590 cm^–1^ corresponding to −OH stretching vibrations. The peaks at 2895 cm^–1^ and 1372 cm^–1^ were assigned to C–H stretching. Additionally, the signals at 1065 cm^–1^, 879 cm^–1^, and 773 cm^–1^ corresponded to a C–O–C pyranose structure and β-glycosidic linkages in pyranose and furanose forms, respectively. These findings were consistent with the standard characteristic FTIR spectrum of AG.^34^ After the addition of gold, the FTIR spectrum of AG–AuNPs showed the appearance of a new peak at 613.87 cm^–1^ corresponding to Au–O stretching vibration (Figure 2b).^35^ The shift in peaks with an increased intensity at 3292 cm^–1^ and decreased flexural vibrations at 1638 cm^–1^ indicated the involvement of hydroxyl groups during AG-AuNPs.

**Figure 2:**
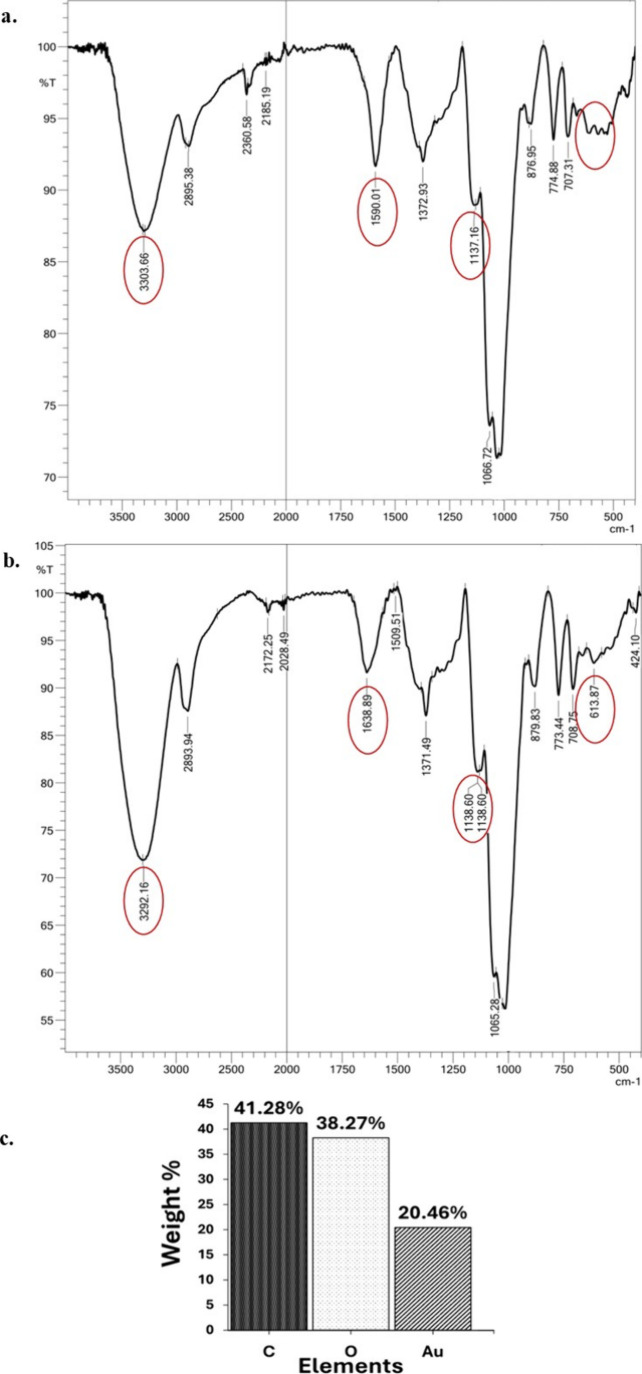
**FTIR and elemental characterization of compounds.** (a, b) FTIR spectra of arabinogalactan and AG-AuNPs. (c) Elemental mapping and relative proportion of carbon, oxygen, and gold in AG-AuNPs.

The position and involvement of different groups in AG-AuNPs formation were confirmed by NMR spectra analysis. The ^1^H NMR spectrum of AG-AuNPs (Figure 3b) was compared with the standard ^1^H NMR spectrum of AG (Figure 3a). The peak δ = 4.12 corresponded to involvement of C2, i.e., H2 proton of arabinofuranose units of AG. Further, the shift in signal at δ = 3.94 and 3.81 might be due to attachment of Au at H6 position of β-D galactopyranose and α-L-arabinofuranose units of AG. The involvement of these groups and their modifications in bond formation was further confirmed using ^13^C NMR. The ^13^C NMR of AG-AuNPs demonstrated a signal δ = 103.36 ppm attributed to a glycosidic bond between the C1 and C6 positions of galactose units of AG (Figure 3c). The appearance of signal δ = 72.71, 70.72, and 68.62 might be due to involvement of C2, C6, and C4 units in AG-AuNPs bonding. The presence of signal at δ = 61.04 suggested that primary hydroxyl groups at the C6 position of β-D galactopyranose might have been substituted with gold atoms during AG-AuNP formation.^32^ These results suggested the ionic interaction between positively charged Au^+^ and negatively charged OH^–^ of AG led to the attachment of AG to the AuNP core. Elemental analysis of AG-AuNPs further confirmed the presence of the following key elements, i.e., carbon (41.28%), oxygen (38.27%), and gold (20.46%), respectively (Figure 2c). Based on these results, the probable structure of AG-AuNPs was elucidated in the figure (Figure 3d).

**Figure 3:**
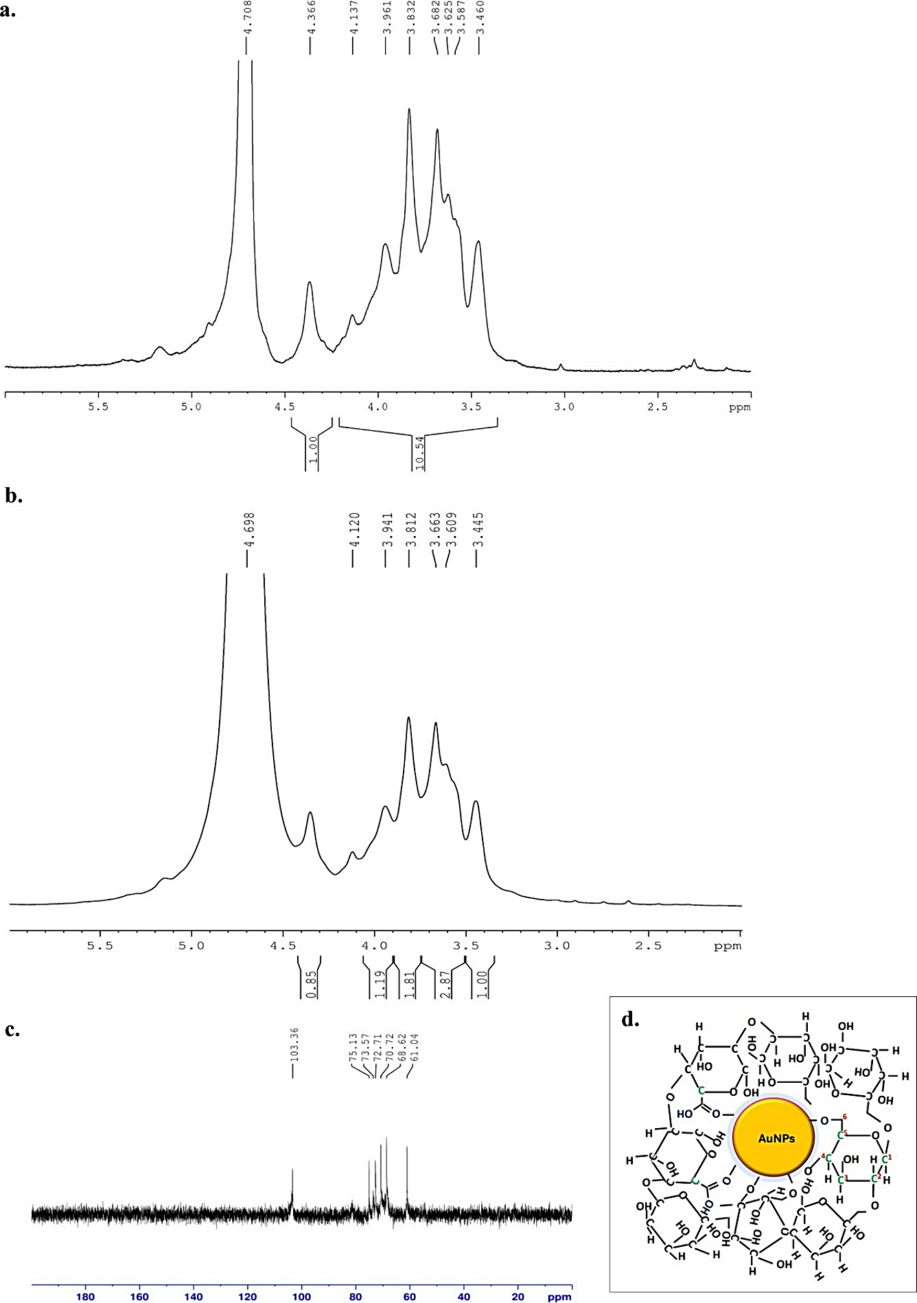
**NMR and probable structure of AG-AuNPs**. (a, b) ^1^H NMR spectra of AG and AG-AuNPs. (c) ^13^C NMR spectrum of AG-AuNPs and (d) Probable structure of AG-AuNPs

### 3.2 AG-AuNPs Demonstrated Good Stability and Favorable Characteristics for *In Vivo* Administration

The stability of AG-AuNPs in serum was confirmed by using UV–visible spectroscopy and hydrodynamic size. The characteristic surface plasmon resonance of AG-AuNPs exhibited a consistent peak at 542 nm at all measured time points (0 to 24 h), indicating minimal aggregation and good stability (Figure 4a). The hydrodynamic size of AG-AuNPs after 24 h of incubation was observed to be 335.8 ± 2.1 nm (Figure 4b). Further, the hemolytic assay revealed that the percentage hemolysis induced by AG-AuNPs at concentrations of 0.5, 1, and 1.5 mg/mL was 2.18%, 3.75%, and 5.6%, respectively (acceptable limit 5%, ASTM E2524) (Figure 4c). These results suggested that AG-AuNPs had good serum stability, and doses less than or equal to 1 mg/mL were suitable for *in vivo* administration.

**Figure 4:**
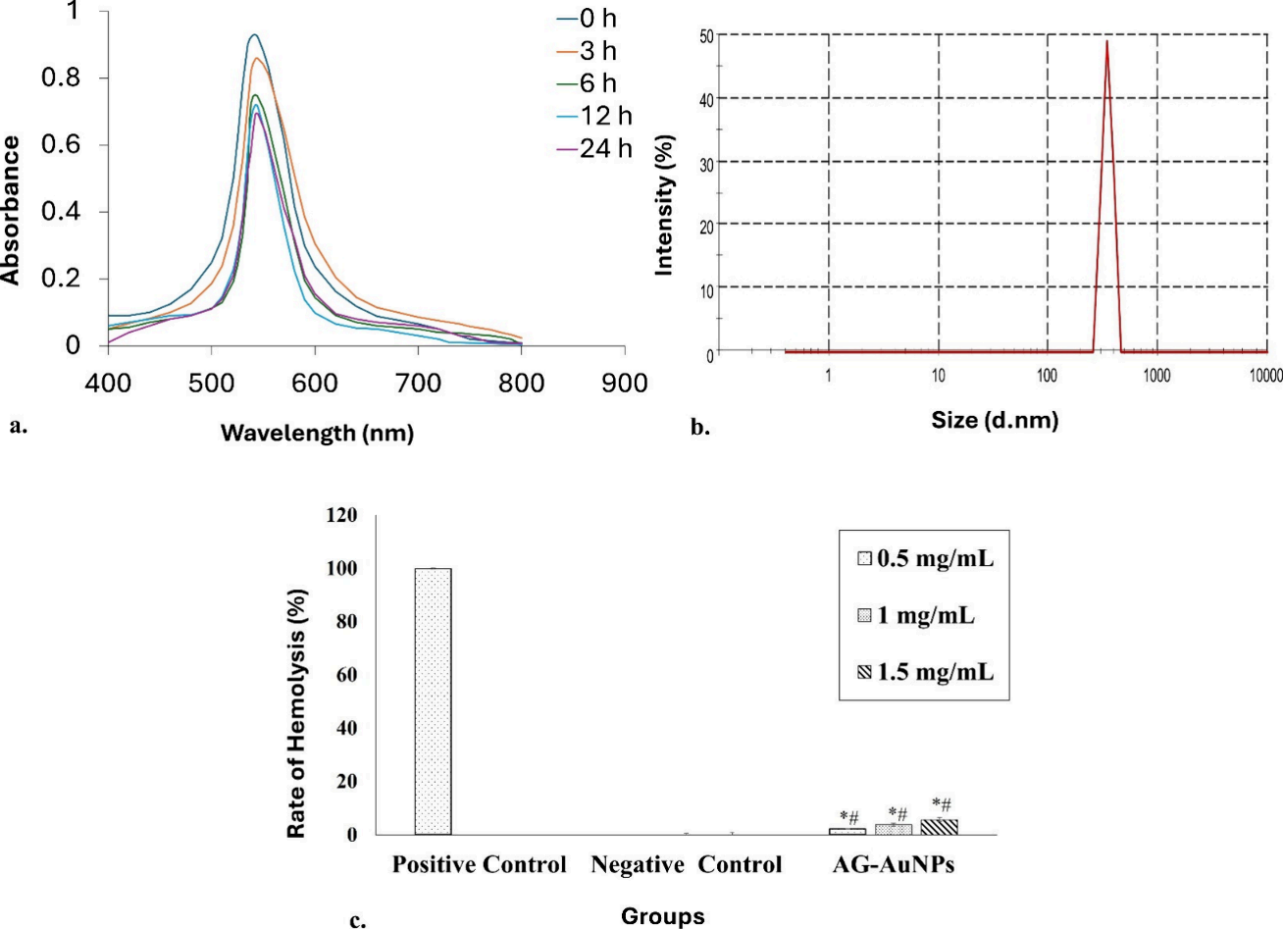
**Stability and hemolysis effect of AG-AuNPs.** (a) UV–vis spectra of AG-AuNPs after incubation in serum at different time intervals between 0 to 24 h. (b) Hydrodynamic size of AG-AuNPs after 24 h of serum incubation. (c) Hemolysis effect of AG-AuNPs at different test concentrations. PBS was used as a negative control, and triton X-100 was used as a positive control. Results were represented as mean ± SD (*n* = 3). * and ^#^ signify *p* ≤ 0.05 as compared to the positive and negative control, respectively.

### 3.3 AG-AuNPs Demonstrated Excellent In Vivo Tumor Localization

The *in vivo* biodistribution study of AG-AuNPs was evaluated in an *N*-nitrosodiethylamine-induced liver cancer model. The concentration of AG-AuNPs accumulated in different organ tissues was quantified using ICP-MS. The localization of AG-AuNPs within liver tumor after 48 h of dose administration was 13.88 ± 0.39 (6.67 ± 0.19% ID/g) significantly higher than normal. Further, tumor to normal [T/N] liver uptake ratio was 1.63 ± 0.15. The AG-AuNPs concentration in other tissues such as lung, spleen, heart, kidney, muscle, blood, testis, and bone marrow were significantly (*p* < 0.01) decreased when compared to liver tumors (Table 1). Furthermore, the tumor to muscle ratio [T/M] was 7.3 ± 0.16, which was significantly (*p* < 0.01) increased when compared to the [T/N] ratio (Supporting Information). This underscores the potential of AG-AuNPs for selective hepatic tumor targeting in HCC treatment.

**Table 1:** Time-Dependent Biodistribution of AG-AuNPs at 48 h of Dose Administration^a^

	48 h	
Tissues	μg Au/g tissues	(%) of given dose
Liver tumor	13.88 ± 0.39	6.67 ± 0.19
Liver	8.53 ± 0.31	4.09 ± 0.15
Lung	3.13 ± 0.14	0.17 ± 0.01
Spleen	3.58 ± 0.20	0.18 ± 0.01
Heart	6.31 ± 0.62	0.52 ± 0.04
Kidney	2.56 ± 0.10	0.4 ± 0.020
Testis	0.53 ± 0.11	0.04 ± 0.01
Muscle	1.93 ± 0.17	0.16 ± 0.01
Blood	0.28 ± 0.01	0.22 ± 0.04

a Data were expressed as mean ± SD and analyzed using one-way ANOVA followed by post hoc test (Tukey’s HSD). *p* ≤ 0.01 was considered statistically significant; Tissue/tumor uptake at 48 h.

### 3.4 AG-AuNPs Demonstrated Strong Anticancer Therapeutic Potential against HCC

The anticancer therapeutic potential of AG-AuNPs was evaluated in the *N-nitrosodiethylamine*-induced liver cancer model. NDEA treatment over a period of 14 weeks led to successful development of hepatic tumors in an *in vivo* rodent model. After 14 weeks, animals with 2-fold increased glypican-3 levels were randomly segregated into cancer and treatment groups. The treatment group (Tumor + AG-AuNPs) received 30 days of AG-AuNPs treatment. After 30 days of treatment, all animals were sacrificed, and liver tissue was excised. The gross morphological observation revealed that animals in the Control and AG-AuNPs groups had normal and distinct liver lobes (Figure 5a,b). However, the liver obtained from animals in Untreated (Tumor group) and treated (Tumor + AG-AuNPs) showed altered morphology and enlarged lobes bearing big (≥3 mm) and small (<3 mm) tumors (Figure 5c,d). The total number of tumors in Tumor and Tumor + AG-AuNPs was 225 and 125, respectively. The tumor multiplicity significantly (*p* ≤ 0.05) decreased to 9.17 ± 0.92 in Tumor + AG-AuNPs group when compared to the Tumor group (16.40 ± 1.12) (Table 2). Further, the histopathological analysis of the Tumor group showed pleomorphic hepatocytes with hyperchromatic nuclei and pseudoglandular pattern, indicating most tumors were at a moderately differentiated stage of HCC (Figure 5g). However, the treatment (Tumor+AG-AuNPs) group showed restored histoarchitecture with uniform nuclei and trabeculae with less chromatin irregularity indicating well-differentiated stage of HCC (Figure 5h). These findings were further supported by dielectric spectroscopy which showed that the Tumor+AG-AuNPs group had 8.4% high conducting tumors when compared to the Tumor group (11.54%), indicating that AG-AuNPs treatment was effective in restoring membrane permeability and ionic concentration.

**Figure 5:**
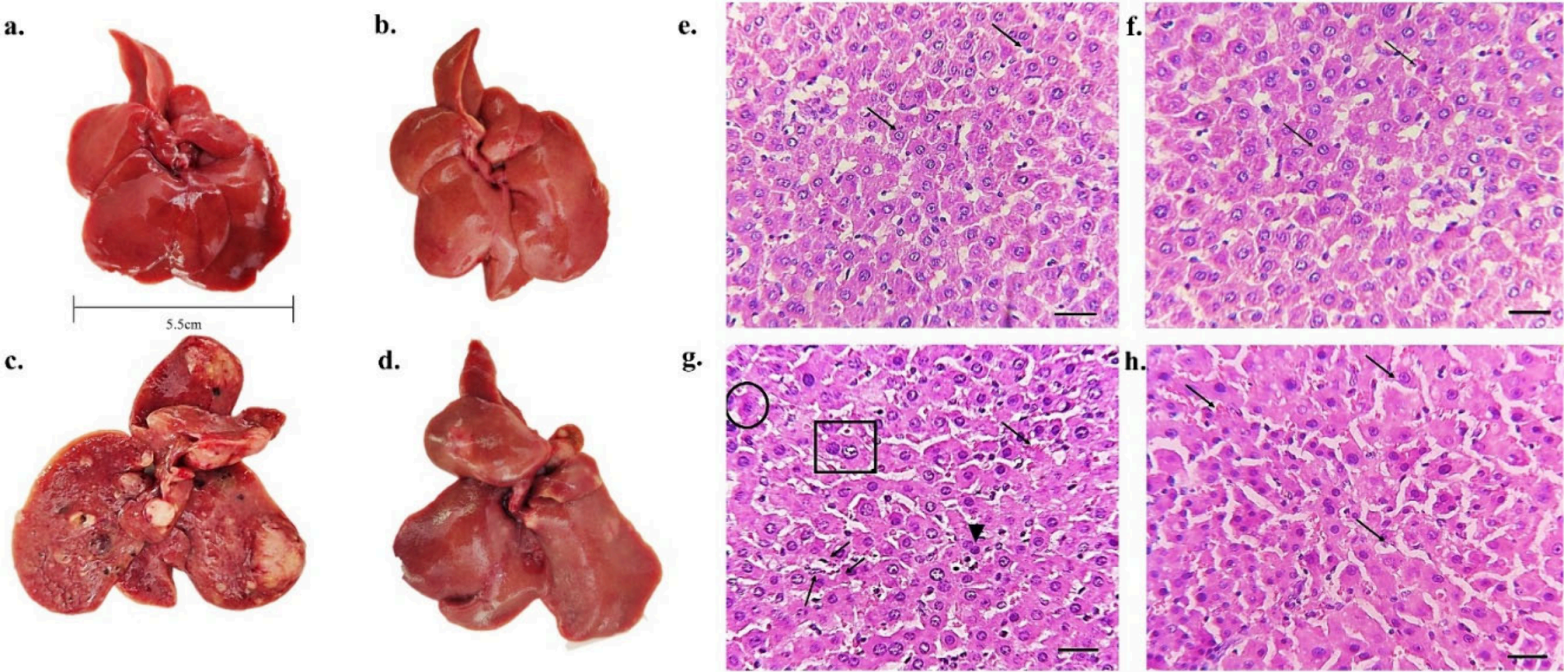
**Gross morphology and histology of liver tissues/tumors after 30 days of treatment period.** (a,b) Control and AG-AuNPs groups showing normal liver morphology with distinct liver lobes. (c) Tumor group showing enlarged liver lobes with multiple firm nodules and neoplastic lesions indicative of aggressive tumors. (d) Tumor + AG-AuNPs group showing relatively clear liver lobular surfaces and less aggressive tumors. (e, f) Photomicrograph of Control & AG-AuNPs group showing histopathology of liver with normal pattern hepatocytes and intact cell membrane (arrows showing normal hepatocytes and sinusoid). (g) Photomicrograph of Tumor group that showed majority of tumors at moderately differentiated stage of HCC (rectangle showing large and hyperchromatic nuclei; arrows showing thickened hepatic trabeculae with pleomorphic hepatocytes, diffuse hemorrhage hyperchromatic nuclei trabecular architecture, and shrinking of nuclei (arrowhead); Circle showing intensely eosinophilic cytoplasm). (h) Tumor + AG-AuNPs group showing tumors at well-differentiated stage of HCC (arrows showing spared hepatocytes and sinusoids) (Magnification: 400× with scale bar 50 μm.

**Table 2:** Tumor Statistics after 30 Days of AG-AuNPs Treatment

Parameter	Control	AG-AuNPs	Tumor^a^	Tumor + AG-AuNPs^a^ ^,^ ^b^ ^,^ ^c^
Hepatosomatic Index	0.02 ± 0.004	0.02 ± 0.005	0.08 ± 0.014*	0.065 ± 0.012*^,#,†^
Big Tumor (≥3 mm)	-	-	88	45
Small Tumor (<3 mm)	-	-	137	80
Tumor Multiplicity	-	-	16.40 ± 1.12	9.17 ± 0.92*
Dielectric Properties (High conducting tumors)	-	-	11.54%	8.4%

a * represents *p* ≤ 0.05 when compared with the Control group.

b # represents *p* ≤ 0.05 when compared with the AG-AuNPs group.

c † represents *p* ≤ 0.05 when compared with Tumor group).

## 4 Discussion

Cancer is an intricate disease whose treatment has become highly challenging and a global health concern.^36^ The limitations posed by conventional therapeutic methods demand further advancements in the development of safe and effective alternative cancer treatment approaches.^37–39^ Nanoparticle-based targeted drug delivery systems have gained huge interest in current research settings. Among different types of nanoparticles, gold nanoparticles (AuNPs) have been extensively exploited in cancer research for the targeted delivery of anticancer agents.^40,41^ However, their functional properties can be further optimized through different surface modification strategies. Among other coating agents, polysaccharides have emerged as promising coating agents with the potential to enhance the therapeutic efficiency of AuNPs.^42^ Various studies have reported that polysaccharide-based AuNPs enhance cancer therapy by synergistically enabling targeted drug delivery, improved cellular uptake, and potent anticancer effects.^43,44^ Among others, arabinogalactan is a naturally occurring highly branched polysaccharide composed of repeating units of galactosyl units that have been widely explored as coating agents for metal-based nanoparticles.^45^ Several reports also suggested that the high affinity of arabinogalactan toward ASGPR makes it a suitable candidate for targeted drug delivery for liver cancer therapy.^46,47^ In addition, several reports on arabinogalactan-coated metal nanoparticles showed that coating not only enhanced surface properties but also contributed to cancer cell death due to its inherent anticancer properties, thus suggesting that arabinogalactan can be a suitable polysaccharide of choice.^48–50^ Therefore, in the present study, AG-AuNPs were synthesized and assessed for their *in vivo* anticancer therapeutic potential against HCC.

The cellular targeting of nanoparticles is primarily dependent on their physicochemical properties like size, shape, charge, composition, and surface modifications as these factors critically influence their cellular uptake and biodistribution. Therefore, in this study, AG-AuNPs were designed to leverage both active and passive targeting mechanisms, ensuring improved therapeutic outcomes for the treatment of hepatocellular carcinoma. Herein, AG-AuNPs were synthesized using the Turkevich method, which is a well-established method designed for the synthesis of gold nanoparticles.^23,33^ AG facilitates the formation of AuNPs through its reducing hydroxyl groups, which convert Au^3+^ to Au^0^ while its polymeric structure acts as a stabilizing agent to prevent aggregation.^51^ The change in the color of reaction mixture from yellow to ruby red indicated the successful synthesis of arabinogalactan-gold nanoparticles (AG-AuNPs). The final pure form of AG-AuNPs was obtained using an ethanol precipitation method. Furthermore, the synthesis of AG-AuNPs was confirmed by using various physicochemical characterization methods. The UV–visible absorption spectrum of AG-AuNPs exhibited an absorption peak with a surface plasmon resonance band at 542 nm. This red-shift absorption band suggested that the synthesized AG-AuNPs might be small spheres with core size ranging from 30 to 50 nm diameter. TEM analysis further confirmed the core size and morphology of AG-AuNPs indicating a mean size of 35.7 ± 2.5 nm with most of the nanoparticles exhibiting a spherical shape. The observed size was well within the expected range, aligning with UV-visible absorption. The hydrodynamic size of AG-AuNPs was 326.1 nm, which was larger as compared to the core size measured by TEM. This difference arises from the inherent distinction between the two characterization techniques where TEM provides information on the dense metallic core of nanoparticles unlike DLS that reflects the hydrodynamic diameter of the particles in colloidal suspension, encompassing not only the metallic core but also the arabinogalactan coating and hydration shell. The other functionalized nanoparticles have also shown similar changes in size, where DLS measurements were consistently larger than the TEM measured core size. The zeta potential analysis further revealed that the surface charge of −22 mV confirmed successful coating of AG onto AuNPs. The negative charge might be attributed to anionic functional groups of arabinogalactan that play an important role in maintaining stable electrostatic interactions and macrophage escape.^52^ These findings suggested that AG-AuNPs with small core size, suitable morphology, stable hydrodynamic size, and negative surface charge enable AG-AuNPs to easily pass through abnormal tumor vasculature with large fenestrations and accumulate in tumor sites via passive targeting thus exhibiting an enhanced permeability and retention (EPR) effect.^53–55^

The XRD analysis further added evidence of structural composition of AG-AuNPs. The appearance of characteristic diffraction peaks confirmed face centered cubic structure of gold.^56^ The presence of an additional peak corresponding to the orthorhombic structure of the saccharides indicated that arabinogalactan was successfully incorporated into the nanoparticles without disrupting the gold lattice. The FTIR spectrum of AG–AuNPs further revealed key bond interactions, confirming successful conjugation of arabinogalactan onto gold nanoparticles. The overall shift in FTIR spectrum of AG-AuNPs with the appearance of new peak at 613.87 cm^–1^ corresponded to a Au–O bond. These findings ensured the successful functionalization of AG with AuNPs. The ^1^H and ^13^C NMR spectra further suggested that AG-AuNPs might have formed by substitution of Au^+^ with negatively charged OH^–^ groups at the C6 position of arabinogalactan backbone. In addition, elemental analysis showing the presence of key elements such as carbon (41.28%), oxygen (38.27%), and gold (20.46%) confirmed successful surface modification of AuNPs with arabinogalactan coating.

Further, to evaluate the suitability of AG-AuNPs for *in vivo* applications, both serum stability and hemolysis-inducing potential of AG-AuNPs were evaluated. The UV–vis spectra of AG-AuNPs in serum demonstrated a consistent characteristic peak at 542 nm throughout 24 h incubation indicating good serum stability. This finding was further supported by an average hydrodynamic size of 335.8 after 24 h of incubation. This small increase in hydrodynamic size might be attributed to the formation of protein corona around the surface of AG-AuNPs. Therefore, the absence of any significant peak broadening and abnormal size confirmed that AG-AuNPs were stable under physiological conditions. Moreover, the hemolysis assay demonstrated that at a specific concentration of 1 mg/mL AG-AuNPs induced less than 5% hemolysis, thereby confirming their suitability for *in vivo* administration (ASTM standard E2524-08).

The structural and surface modifications play a crucial role in determining the biological fate of AG-AuNPs. Therefore, an *in vivo* biodistribution study was conducted in *N-nitrosodiethylamine (NDEA)*-induced liver cancer model. ICP-MS analysis revealed significantly higher accumulation of AG-AuNPs in liver tumors (13.88 ± 0.39 μg/g) after 48 h of intravenous administration compared to other tissues. The amount of AG-AuNPs in the blood was observed to be 0.28 ± 0.01 μg/g after 48 h. Similar findings were reported in previous studies where biodistribution of PEG–Gal-coated gold nanoparticles (ASGPR-targeted) were compared with nonreceptor-specific citrate-coated gold nanoparticles in an *in vivo* rodent model.^57^ The preferential tumor accumulation in the present study was evident from the observation where tumors had 1.6 times higher AG-AuNPs uptake compared to nontumor liver tissue. This selective localization in liver emphasized the impact of arabinogalactan functionalization on nanoparticle biodistribution at the therapeutic level. Moreover, the tumor to muscle ratio was also significantly higher. This enhanced targeting efficiency could be attributed to the dual targeting mechanism for AG-AuNPs, i.e., active and passive tumor targeting. Passive targeting is primarily driven by an ‘enhanced permeability and retention effect’ where leaky vasculature and inefficient lymphatic drainage of tumors facilitate the accumulation of AG-AuNPs within a tumor microenvironment.^58^ Meanwhile, active targeting relies on high binding affinity of arabinogalactan for ASGP receptors present on hepatoma cells, leading to ASGPR-mediated endocytosis.^59^ The dual targeting strategy collectively might have contributed to efficient uptake of AG-AuNPs, enhanced tumor retention, and improved therapeutic effect. Considering the excellent tumor uptake and favorable biodistribution of AG-AuNPs we further investigated their *in vivo* anticancer therapeutic potential.

The anticancer therapeutic potential of AG-AuNPs was evaluated in a NDEA-induced liver cancer model. Following 14 weeks of NDEA exposure, the development of the liver cancer model was validated through assessment of serum glypican-3, an early serum marker for liver cancer. The animals demonstrating a 2-fold or more increase in GPC-3 levels relative to Control animals were considered positive for liver cancer and divided into Tumor and Tumor + AG-AuNPs groups. After 30 days of AG-AuNPs treatment, the decrease in the total number of liver tumors and tumor multiplicity in Tumor + AG-AuNPs group as compared to Tumor group demonstrated anticancer therapeutic potential of AG-AuNPs. The histological findings provided further evidence of the therapeutic potential of AG-AuNPs in mitigating liver cancer. The untreated tumor group exhibited characteristic features of ″moderately differentiated HCC” unlike the treatment group that showed lower-grade tumors with most tumors at a ‘well-differentiated’ stage. These findings suggested that reduction in tumor number could be due to induction of ferroptosis.^60^ Upon internalization, AG-AuNPs directly interact with ferritin through electrostatic forces and surface binding that destabilize its quaternary structure leading to subsequent release of Fe^2+^ into the labile iron pool.^61^ The elevated Fe^2+^ undergoes Fenton’s reaction producing highly reactive radicals resulting in lipid peroxidation of biological membranes. The lipid peroxidation of mitochondrial membrane alters mitochondrial functioning eventually resulting in ferroptosis.^62,63^ Furthermore, the AG-AuNPs-treated group exhibited significantly decreased high-conducting tumors in comparison to the untreated Tumor group. These findings were in accordance with previous studies indicated that tumor tissues exhibited higher conductivity due to abnormal angiogenesis, increased metabolic activity, and perturbations in the ion transport mechanism.^64^ These results highlighted the potential of AG-AuNPs as an effective strategy for the treatment of liver cancer.

Overall, AG-AuNPs demonstrated good anticancer therapeutic potential against HCC. This was well-evidenced in our results, where AG-AuNPs treatment led to selective tumor localization and marked reduction in tumor number and tumor multiplicity. These findings suggest that AG-AuNPs might be a potential targeted anticancer therapeutic agent for the treatment of HCC.

## Supplementary Material



## Data Availability

Data are available and can be provided upon a request to the corresponding author.

## Notes

In vivo biodistribution of AG-AuNPs in an HCC rodent model (Table S1) (PDF)

## References

[1] Ottaiano A., Ianniello M., Santorsola M., Ruggiero R., Sirica R., Sabbatino F., Perri F., Cascella M., Di Marzo M., Berretta M., Caraglia M., Nasti G., Savarese G. (2023). From chaos
to opportunity: Decoding cancer heterogeneity for enhanced treatment
strategies. Biology.

[2] Liu B., Zhou H., Tan L., Siu K. T. H., Guan X. Y. (2024). Exploring
treatment options in cancer: Tumor treatment strategies. Signal Transduction and Targeted Therapy.

[3] Cosma M., Mocan T., Sabau L. I., Pop T., Mosteanu O., Mocan L. A. (2025). Narrative review on functionalized
nanoparticles for
the treatment and early detection of hepatocellular carcinoma. Applied Sciences.

[4] Chehelgerdi M., Chehelgerdi M., Allela O. Q. B., Pecho R. D. C., Jayasankar N., Rao D. P., Thamaraikani T., Vasanthan M., Viktor P., Lakshmaiya N., Saadh M. J., Amajd A., Abo-Zaid M. A., Castillo-Acobo R. Y., Ismail A. H., Amin A. H., Akhavan-Sigari R. (2023). Progressing nanotechnology to improve targeted cancer
treatment: Overcoming hurdles in its clinical implementation. Molecular Cancer.

[5] Bloise N., Strada S., Dacarro G., Visai L. (2022). Gold nanoparticles
contact with cancer cell: A brief update. International
Journal of Molecular Sciences.

[6] Deivayanai V. C., Thamarai P., Karishma S., Saravanan A., Yaashikaa P. R., Vickram A. S., Hemavathy R. V., Kumar R. R., Rishikesavan S., Shruthi S. (2024). A comprehensive review
on advances in nanoparticle-mediated cancer therapeutics: Current
research and future perspectives. Cancer Pathogenesis
and Therapy.

[7] Aljarba N. H., Imtiaz S., Anwar N., Alanazi I. S., Alkahtani S. (2022). Anticancer
and microbial activities of gold nanoparticles: A mechanistic review. Journal of King Saud University - Science.

[8] Martínez-Torres A. C., Zarate-Triviño D. G., Lorenzo-Anota H. Y., Ávila-Ávila A., Rodríguez-Abrego C., Rodríguez-Padilla C. (2018). Chitosan gold nanoparticles induce
cell death in Hela and MCF-7 cells through reactive oxygen species
production. Int. J. Nanomed..

[9] Arcos
Rosero W. A., Bueno Barbezan A., Daruich de Souza C., Chuery Martins Rostelato M. E. (2024). Review of advances in coating and
functionalization of gold nanoparticles: From theory to biomedical
application. Pharmaceutics.

[10] Leng M., Jiang H., Zhang S., Bao Y. (2024). Green synthesis of
gold nanoparticles from polygahatous polysaccharides and their anticancer
effect on hepatic carcinoma through immunoregulation. ACS Omega.

[11] Zhu C. D., Zheng Q., Wang L. X., Xu H. F., Tong J. L., Zhang Q. A., Wan Y., Wu J. Q. (2015). Synthesis of novel
galactose functionalized gold nanoparticles and its radiosensitizing
mechanism. J. Nanobiotechnol..

[12] Song M., Aipire A., Dilxat E., Li J., Xia G., Jiang Z., Fan Z., Li J. (2024). Research progress
of
polysaccharide-gold nanocomplexes in drug delivery. Pharmaceutics.

[13] Pfeifer L., Baumann A., Petersen L. M., Höger B., Beitz E., Classen B. (2021). Degraded arabinogalactans and their
binding properties to cancer-associated human galectins. International Journal of Molecular Sciences.

[14] Guo R., Chen M., Ding Y., Yang P., Wang M., Zhang H., He Y., Ma H. (2022). Polysaccharides as
potential anti-tumor biomacromolecules: A review. Frontiers in Nutrition.

[15] Samra Y. A., Abdelghany A. M., Zaghloul R. A. (2021). Polydatin Gold Nanoparticles Potentiate
Antitumor Effect of Doxorubicin in Ehrlich Ascites Carcinoma-Bearing
Mice. J. Biochem Mol. Toxicol.

[16] Kattumuri V., Katti K., Bhaskaran S., Boote E. J., Casteel S. W., Fent G. M., Robertson D. J., Chandrasekhar M., Kannan R., Katti K. V. (2007). Gum arabic as a phytochemical construct
for the stabilization of gold nanoparticles: In vivo pharmacokinetics
and X-ray-contrast-imaging studies. Small.

[17] Lin C. M., Kao W. C., Yeh C. A., Chen H. J., Lin S. Z., Hsieh H. H., Sun W. S., Chang C. H., Hung H. S. (2015). Hyaluronic
acid-fabricated nanogold delivery of the inhibitor of apoptosis protein-2
siRNAs inhibits benzo­[a]­pyrene-induced oncogenic properties of lung
cancer A549 cells. Nanotechnology.

[18] Joseph M. M., Aravind S. R., Varghese S., Mini S., Sreelekha T. T. (2013). PST-gold
nanoparticle as an effective anticancer agent with immunomodulatory
properties. Colloids Surf., B.

[19] Kumar P., Ashique S., Sharma H., Yasmin S., Islam A., Mandal S., Gowda B. H. J., Khalid M., Ansari M. Y., Singh M., Ehsan I., Taj T., Taghizadeh-Hesary F. (2025). A narrative
review on the use of green synthesized metallic nanoparticles for
targeted cancer therapy. Bioorganic Chemistry.

[20] Yang L. C., Lai C. Y., Hsieh C. C., Lin W. C. (2019). Natural killer cell-mediated
anticancer effects of an arabinogalactan derived from rice hull in
CT26 colon cancer-bearing mice. Int. J. Biol.
Macromol..

[21] Pathak P., Dhawan V., Magarkar A., Danne R., Govindarajan S., Ghosh S., Steiniger F., Chaudhari P., Gopal V., Bunker A., Róg T., Fahr A., Nagarsenker M. (2016). Design of cholesterol arabinogalactan
anchored liposomes for asialoglycoprotein receptor mediated targeting
to hepatocellular carcinoma: In silico modeling, in vitro and in vivo
evaluation. Int. J. Pharm..

[22] D’Souza A. A., Devarajan P. V. (2015). Asialoglycoprotein
receptor mediated hepatocyte targeting
- strategies and applications. J. Controlled
Release.

[23] Dong J., Carpinone P. L., Pyrgiotakis G., Demokritou P., Moudgil B. M. (2020). Synthesis of precision gold nanoparticles using Turkevich
method. KONA Powder and Particle Journal.

[24] Foo Y. (2017). *Curcuma mangga*-mediated synthesis of gold nanoparticles:
Characterization, stability, cytotoxicity, and blood compatibility. Nanomaterials.

[25] Neun B. W., Ilinskaya A. N., Dobrovolskaia M. A. (2018). Updated method for in vitro analysis
of nanoparticle hemolytic properties. Methods
Mol. Biol..

[26] Jilkova Z. M., Kuyucu A. Z., Kurma K., Ahmad Pour S. T., Roth G. S., Abbadessa G., Yu Y., Schwartz B., Sturm N., Marche P. N., Hainaut P., Decaens T. (2018). Combination
of AKT inhibitor ARQ 092 and sorafenib potentiates inhibition of tumor
progression in cirrhotic rat model of hepatocellular carcinoma. Oncotarget.

[27] Bailly A. L., Correard F., Popov A., Tselikov G., Chaspoul F., Appay R., Al-Kattan A., Kabashin A. V., Braguer D., Esteve M. A. (2019). In vivo evaluation
of safety, biodistribution and pharmacokinetics
of laser-synthesized gold nanoparticles. Sci.
Rep..

[28] Zhang Y., Liu A. T., Cornejo Y. R., van Haute D., Berlin J. M. (2020). A systematic comparison of in vitro
cell uptake and
in vivo biodistribution for three classes of gold nanoparticles with
saturated PEG coatings. PLoS One.

[29] Sonavane G., Tomoda K., Makino K. (2008). Biodistribution
of colloidal gold
nanoparticles after intravenous administration: Effect of particle
size. Colloids Surf., B.

[30] Shetty S., Anushree U., Kumar R., Bharati S. (2020). Electrical conductivity
spectra of hepatic tumors reflect hepatocellular carcinoma progression
in mice. Biomedical Physics & Engineering
Express.

[31] Cardiff R. D., Miller C. H., Munn R. J. (2014). Manual hematoxylin and eosin staining
of mouse tissue sections. Cold Spring Harbor
Protocols.

[32] D’Souza A. A., Jain P., Galdhar C. N., Samad A., Degani M. S., Devarajan P. V. (2013). Comparative in silico-in vivo evaluation of ASGP-R
ligands for hepatic targeting of curcumin Gantrez nanoparticles. AAPS Journal.

[33] Khorsand
Zak A., Razali R., Abd Majid W. H., Darroudi M. (2011). Synthesis and characterization
of a narrow size distribution of zinc oxide nanoparticles. Int. J. Nanomed..

[34] Wu J., Xu Y., Zhu B., Liu K., Wang S., Sheng Y., Wang H., Shi S., Zhang Q., Wang S., Qin L. (2020). Characterization of an arabinogalactan from the fruit hulls of *Ficus pumila* Linn. and its immunomodulatory effect. Journal of Functional Foods.

[35] Barnawi N., Allehyani S., Seoudi R. (2022). Biosynthesis and characterization
of gold nanoparticles and its application in eliminating nickel from
water. Journal of Materials Research and Technology.

[36] Biemar F., Foti M. (2013). Global progress against
cancer - challenges and opportunities. Cancer
Biology & Medicine.

[37] Chakraborty S., Rahman T. (2012). The difficulties in
cancer treatment. ecancermedicalscience.

[38] Debela, D. T. ; Muzazu, S. G. Y. ; Heraro, K. D. ; Ndalama, M. T. ; Mesele, B. W. ; Haile, D. C. ; Kitui, S. K. ; Manyazewal, T. New approaches and procedures for cancer treatment: Current perspectives. SAGE Open Medicine 2021, 9. 10.1177/20503121211034366 PMC8366192 34408877

[39] Deng G. L., Zeng S., Shen H. (2015). Chemotherapy
and target therapy for
hepatocellular carcinoma: New advances and challenges. World Journal of Hepatology.

[40] Dadwal A., Baldi A., Kumar Narang R. (2018). Nanoparticles
as carriers for drug
delivery in cancer. Artificial Cells, Nanomedicine,
and Biotechnology.

[41] Yao Y., Zhou Y., Liu L., Xu Y., Chen Q., Wang Y., Wu S., Deng Y., Zhang J., Shao A. (2020). Nanoparticle-based drug delivery
in cancer therapy and its role in
overcoming drug resistance. Frontiers in Molecular
Biosciences.

[42] Alzahrani A. R., Ibrahim I. A. A., Shahzad N., Shahid I., Alanazi I. M., Falemban A. H., Azlina M. F. N. (2023). An application
of carbohydrate polymers-based
surface-modified gold nanoparticles for improved target delivery to
liver cancer therapy - a systemic review. Int.
J. Biol. Macromol..

[43] Liu H., Gu L., Ye Y., Zhang M. (2024). *Auricularia auricula* polysaccharide-mediated
green synthesis of highly stable Au NPs. Polysaccharides.

[44] Chen X., Zhao X., Gao Y., Yin J., Bai M., Wang F. (2018). Green synthesis of gold nanoparticles using carrageenan oligosaccharide
and their in vitro antitumor activity. Marine
Drugs.

[45] Fincher G.
B., Stone B. A., Clarke A. E. (1983). Arabinogalactan-proteins: Structure,
biosynthesis, and function. Annual Review of
Plant Physiology.

[46] Yousef S., Alsaab H. O., Sau S., Iyer A. K. (2018). Development of asialoglycoprotein
receptor directed nanoparticles for selective delivery of curcumin
derivative to hepatocellular carcinoma. Heliyon.

[47] Pranatharthiharan S., Patel M. D., Malshe V. C., Pujari V., Gorakshakar A., Madkaikar M., Ghosh K., Devarajan P. V. (2017). Asialoglycoprotein
receptor targeted delivery of doxorubicin nanoparticles for hepatocellular
carcinoma. Drug Delivery.

[48] Kolovskaya O. S., Zamay T. N., Zamay G. S., Babkin V. A., Medvedeva E. N., Neverova N. A., Kirichenko A. K., Zamay S. S., Lapin I. N., Morozov E. V., Sokolov A. E., Narodov A. A., Fedorov D. G., Tomilin F. N., Zabluda V. N., Alekhina Y., Lukyanenko K. A., Glazyrin Y. E., Svetlichnyi V. A., Berezovski M. V., Kichkailo A. S. (2020). Aptamer-conjugated superparamagnetic
ferroarabinogalactan
nanoparticles for targeted magnetodynamic therapy of cancer. Cancers (Basel).

[49] Tang S., Wang T., Jiang M., Huang C., Lai C., Fan Y., Yong Q. (2019). Construction of arabinogalactans/selenium nanoparticles
composites for enhancement of the antitumor activity. Int. J. Biol. Macromol..

[50] Aleksandrova G. P., Gasilova E. R. (2025). Comparison of core-shell
colloids of silver and gold
nanoparticles capped by arabinogalactan. Particle
& Particle Systems Characterization.

[51] Gasilova E. (2012). Colloids of gold nanoparticles
protected from aggregation with arabinogalactan. Macromol. Symp..

[52] Udupi A., Shetty S., Aranjani J. M., Kumar R., Bharati S. (2025). Anticancer
therapeutic potential of multimodal targeting agent - “phosphorylated
galactosylated chitosan coated magnetic nanoparticles” against
N-nitrosodiethylamine-induced hepatocellular carcinoma. Drug Delivery and Translational Research.

[53] Lazarovits J., Chen Y. Y., Sykes E. A., Chan W. C. W. (2015). Nanoparticle-blood
interactions: The implications on solid tumour targeting. Chem. Commun..

[54] Zein R., Sharrouf W., Selting K. (2020). Physical properties
of nanoparticles
that result in improved cancer targeting. Journal
of Oncology.

[55] Kalyane D., Raval N., Maheshwari R., Tambe V., Kalia K., Tekade R. K. (2019). Employment of enhanced
permeability and retention effect
(EPR): Nanoparticle-based precision tools for targeting of therapeutic
and diagnostic agents in cancer. Materials Science
and Engineering C.

[56] Lee C. F., Chang C. L., Yang J. C., Lai H. Y., Chen C. H. (2012). Morphological
determination of face-centered-cubic metallic nanoparticles by X-ray
diffraction. J. Colloid Interface Sci..

[57] Ding Y., Liang J. J., Geng D. D., Wu D., Dong L., Shen W. B., Xia X. H., Zhang C. (2014). Development of a liver-targeting
gold-PEG-galactose nanoparticle platform and a structure-function
study. Particle & Particle Systems Characterization.

[58] Subhan M. A., Yalamarty S. S. K., Filipczak N., Parveen F., Torchilin V. P. (2021). Recent
Advances in Tumor Targeting via Epr Effect for Cancer Treatment. Journal of Personalized Medicine.

[59] Warrier D. U., Dhanabalan A. K., Krishnasamy G., Kolge H., Ghormade V., Gupta C. R., Ambre P. K., Shinde U. A. (2022). Novel derivatives
of arabinogalactan, pullulan and lactobionic acid for targeting asialoglycoprotein
receptor: Biomolecular interaction, synthesis and evaluation. Int. J. Biol. Macromol..

[60] Fernandez-Acosta R., Iriarte-Mesa C., Alvarez-Alminaque D., Hassannia B., Wiernicki B., Diaz-Garcia A. M., Vandenabeele P., Vanden Berghe T., Pardo Andreu G. L. (2022). Novel iron oxide nanoparticles induce
ferroptosis in a panel of cancer cell lines. Molecules.

[61] Chittineedi P., Mohammed A., Abdul Razab M. K. A., Mat Nawi N., Pandrangi S. L. (2023). Polyherbal
formulation conjugated to gold nanoparticles induced ferroptosis in
drug-resistant breast cancer stem cells through ferritin degradation. Frontiers in Pharmacology.

[62] Ou R., Aodeng G., Ai J. (2023). Advancements in the application of
the Fenton reaction in the cancer microenvironment. Pharmaceutics.

[63] Endale H. T., Tesfaye W., Mengstie T. A. (2023). ROS-induced lipid
peroxidation and
their role in ferroptosis. Frontiers in Cell
and Developmental Biology.

[64] Lee R., Lee S. M., Kim H. J., Kim S. Y., Son M., Song J. H., Lkhamsuren K., Park I. H., Choi I. H., Park Y. N., Shin J. S., Yoo K. H. (2017). Dielectric imaging
for differentiation between cancer and inflammation in vivo. Sci. Rep..

