# Supplementary material for: Arabinogalactan-Functionalized Gold Nanoparticles Demonstrated Remarkable Anticancer Therapeutic Effect against Hepatocellular Carcinoma
Source: Mol Pharm. 2025 Oct 13;22(11):6646–57. doi: 10.1021/acs.molpharmaceut.5c00598 (PMC12587396; doi:10.1021/acs.molpharmaceut.5c00598)
Supplement: Supplementary file 1 [file mp5c00598_si_001.pdf]

Arabinogalactan functionalized gold nanoparticles demonstrated remarkable anticancer therapeutic effect against hepatocellular carcinoma

Vasumathi R[1], Maya P Shetty[1], Suvalakshmi.S[1], Revathi P Shenoy[2], Srinivas Mutalik[3], Sanjay Bharati[1]*

[1]Department of Nuclear Medicine, Manipal College of Health Professions, Manipal Academy of Higher Education (MAHE), Manipal 576104, Karnataka, India.

[2]Department of Biochemistry, Kasturba Medical College, Manipal Academy of higher education, Manipal Academy of Higher Education (MAHE), Manipal 576104, Karnataka, India.

[3]Department of Pharmaceutics, Manipal College of Pharmaceutical Sciences, Manipal Academy of Higher Education (MAHE), Manipal 576104, Karnataka, India.

Supporting Information

S1. *In vivo* biodistribution of AG-AuNPs in HCC rodent model

Table S1. Tumor to organ ratio of percentage of Ag-AuNPs distributed in different tissues at 48 h post injection

Organ	Tumor to organ ratio of AG-AuNPs
	48h
**Blood [T/B]**	49.5 ± 0.34
**Heart [T/H]**	2.2 ± 0.14
**Lung [T/L]**	4.43 ± 0.29
**Liver [T/N]**	1.63 ± 0.15
**Muscle [T/M]**	7.3 ± 0.16
**Kidney [T/K]**	3.85 ± 0.16
**Testis [T/T]**	26.18 ± 0.11
**Spleen [T/S]**	3.88 ± 0.42
**Bone marrow [T/B.M]**	138.78 ± 0.13

(Data were expressed as mean ± SD and analyzed using one-way ANOVA followed by post hoc test (Tukey's HSD) $p \leq 0.01$)
